# The Current State of Undergraduate Trauma and Orthopedics Training in Saudi Arabia: A Survey-Based Study of Sixth-Year Medical Students’ and Interns’ Learning Experience and Subjective Clinical Competence

**DOI:** 10.7759/cureus.39974

**Published:** 2023-06-05

**Authors:** Bandar M Hetaimish, Osamah Abualross, Abdulrahman Alesawi, Mohammed Namenkani, Abdulaziz Alramadhani, Ramy Samargandi

**Affiliations:** 1 Orthopedic Surgery, Faculty of Medicine, University of Jeddah, Jeddah, SAU; 2 Medicine, Faculty of Medicine, University of Jeddah, Jeddah, SAU; 3 Orthopedic Surgery, CHRU de Tours, Tours, FRA

**Keywords:** training, curriculum, undergraduate, medical education, orthopedics

## Abstract

Background and objective

Students frequently complain about the lack of practical skill learning and the poor quality of the medical school curriculum. In light of this, the purpose of this study was to assess the learning experience and subjective clinical competence of final-year medical students and interns in the field of orthopedics in Saudi Arabia (SA).

Methods

A cross-sectional observational descriptive study utilizing an electronically validated survey was conducted, which included the following six main sections: introduction, demographics, self-assessment of competency regarding certain orthopedic skills, clinical experience in orthopedics, orthopedics curriculum assessment, and choice of future career specialty.

Results

The total number of participants was 794. Among them, 33% (n=160) and 37.1% (180) had attended no "trauma meetings" or "operating room (OR)" sessions respectively, and only 21.9% (n=106) had attended more than five clinics. Subjective competence in history taking was highest (mean: 8.925 ±1.299) among students who had received more than four weeks of orthopedic rotation and attended more than six clinics. The students who had completed more than four weeks of orthopedic rotation and more than six bedside sessions scored the highest in terms of subjective competence in handling orthopedic patients in primary care settings (mean: 8.014 ±1.931).

Conclusion

The survey indicates that the amount of orthopedic training provided by institutions varies, with some students receiving less training than recommended. However, longer rotations lead to greater perceived orthopedic competence. Students and interns with more exposure to orthopedics through curriculum and elective rotations demonstrated a greater interest in pursuing orthopedics as a future career.

## Introduction

Musculoskeletal (MSK) complaints are among the primary reasons why individuals seek medical attention in the United States of America (USA), comprising 15-30% of all primary care encounters [1]. Musculoskeletal disorders (MSDs) are on the rise in Saudi Arabia (SA) as well and it is currently the third leading cause of hospitalization in the country; in 2020, a total of 25,561 ​cases of injuries from road traffic accidents (RTA) resulted in ​4,618 deaths [2,3]. Furthermore, a study of global injury morbidity and mortality from 1990 to 2017 reported that Saudi Arabia recorded a "years of life lost" (YLL) rate of more than 30% due to injuries [4].

A survey-based study conducted in the United Kingdom (UK) assessing the quality and duration of trauma and orthopedics training in medical schools in the UK has demonstrated that 19.3% of students had not received placement in orthopedics, and the mean duration of their placements was 2.5 weeks; moreover, 37.4% described their training as "poor" [5]. Another UK study indicates that medical schools may not be doing enough to guarantee that medical students gain a fundamental understanding of MSK medicine [6]. Locally, according to Alrwaily et al. [2], Saudi Arabian medical students, interns, and general healthcare practitioners appear to lack adequate knowledge of the MSK system.

Students frequently complain about the lack of practical skill learning and the poor quality of medical school curricula, and there is a disconnect between physician expertise in MSK medicine and the prevalence of MSDs [7,8]. Therefore, we believe all medical school graduates must have a fundamental understanding of MSK problems. Furthermore, according to multiple studies, the lack of sufficient orthopedic surgery training throughout medical school in addition to a dearth of elective orthopedic lectures, MSK education, and formal mentorship opportunities have all been linked to altering students' interest in application to orthopedic residencies [8,9]. Hence, the purpose of this study is to evaluate the quality of orthopedic training received by Saudi Arabian medical students, correlate it with their competency in MSK medicine, and analyze how these factors determine their eventual choice of specialty.

## Materials and methods

Study design

This was an observational descriptive cross-sectional study.

Eligibility and participants

Samples were taken randomly by using a convenience sampling technique from our target population of enrolled final-year (sixth-year) medical students and interns at the time when the study was conducted, from a total of 29 different universities. Our inclusion criteria were as follows: active MBBS undergraduate final-year medical students and interns at all public and private medical schools in Saudi Arabia.

Sample size

The Raosoft calculator (Raosoft Inc, Seattle, WA) was used to determine the sample size. Based on recent data, we calculated the sample size and estimated that there were 14,000 medical students and interns in Saudi Arabia. To obtain a 95% confidence interval with a 5% margin of error, 374 samples were required. The sample size was increased as it was anticipated that fewer respondents would complete an online survey.

Setting and sources

The survey was filled out by the participants on Google Forms (Alphabet Inc., Mountain View, CA). The questionnaire was created and validated based on previous studies with the permission of the authors [5].

The questionnaire consisted of the following six sections: introduction, demographics, self-assessment of competency regarding some orthopedic skills, clinical experience in orthopedics (e.g., summer electives) or previous qualifications, orthopedics curriculum assessment, and future career specialty. Age, gender, and university were among the demographic variables assessed. A few terms in the original questionnaire were replaced with appropriate synonyms to make it fit the medical education system in Saudi Arabia. It was made mandatory to complete/fill in all items in the questionnaire to reduce the number of incomplete entries. The types of responses were binary (yes/no), multiple choice, multiple grids, Likert scale (1-10), or free text, depending on the question.

The data collection was undertaken in a period of two weeks in October 2022. The learning experience and subjective clinical competence were assessed. Several factors such as gender, interest, summer electives, and clinical exposure were investigated with regard to future specialty choice.

Ethical consideration

The Bioethics Committee of Scientific and Medical Research at the University of Jeddah approved the study (UJ-REC-079). Consent was obtained from each participant before starting the electronic questionnaire and it was mandatory to provide it to be included in the study.

Statistical analysis

Data were entered into Microsoft Excel. It was then transferred to and analyzed using IBM SPSS Statistics for Windows, Version 20.0 (IBM Corp., Armonk, NY). Quantitative variables are presented as mean and standard deviation (SD) while qualitative variables are presented as frequencies and percentages. Chi-square or Fisher's exact test was used for the comparison of two categorical variables where appropriate. An independent t-test was used for the comparison of categorical with continuous variables. Multiple bar graphs were used for the graphical representation of mean learning differences on the basis of orthopedic rotation. The participants were successively split into the groups with the highest and lowest perceived levels of competence using a variable in the tree diagram, and then each subgroup was divided once again until no more statistically meaningful splits were possible. Multiple tree diagrams were utilized to demonstrate the relationship between the factors that determined the competency of students based on a predetermined set of dependent factors (e.g., history taking in orthopedics, presenting examination findings, interpreting and presenting radiographs, management of orthopedics patients in primary care setting).

## Results

Demographics

**Figure 1:**
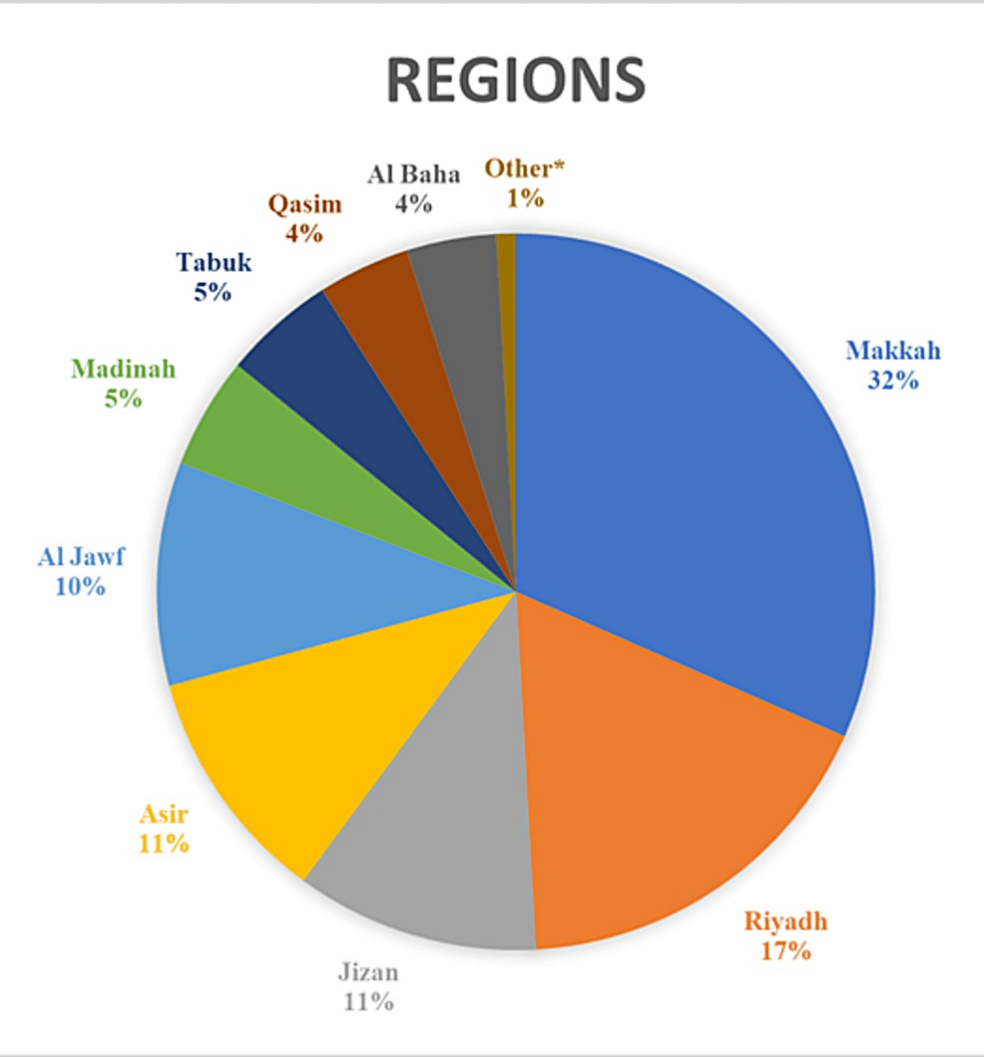
Pie chart depicting participants per region *Includes regions with a participant count of less than 1%

The total number of participants who completed the online survey was 794. A total of 29 universities/colleges across 13 different regions in SA were included (Figure 1); 45.5% (n=361) of respondents were male, while 54.5% (n=433) were female, and the mean age of the participants was 24.3 ±1.72 years. Of note, 95.3% of respondents (n=757) stated that they had no prior undergraduate education or training before beginning medical school. Additionally, only 19.9% (158) of students had undertaken a summer elective in orthopedics, with "3-4 weeks" being the most commonly cited duration of the program (n=60, 7.6%) (Table 1).

**Table 1:** Demographic characteristics of the participants

Variable	N (%)				
Gender					
Male		361 (45.5%)			
Female		433 (54.5%)			
Age					
<24 years		254 (32%)			
25-26 years		378 (47.6%)			
>26 years		162 (20.4%)			
Prior undergraduate qualifications					
Yes		37 (4.7%)			
No		757 (95.3%)			
Presence of orthopedic rotation in the curriculum					
Yes		485 (61.1%)			
No		309 (38.9%)			
Undertaken a summer elective in orthopedics					
Yes (duration in weeks)		158 (19.9%)			
		N=13, 1.6% (<1)	N=33, 4.2% (1-2)	N=60, 7.6% (3-4)	N=52, 6.5% (>4)
No		636 (80.1%)			

Curriculum assessment

Of note, 61.1% (n=485) of students responded "yes" when asked if their medical school provided an orthopedic rotation in their curriculum. The most common duration of rotations was ">4 weeks" (32.8%, n=159), and 54.6% (n=265) of students received ">10" lectures. Regarding bedside/informal group sessions, clinics, operating room (OR) assembly, and trauma meetings, "1-5 sessions" was the most commonly reported answer: 56.9% (n=276), 57.1% (n=277), 48% (n=233), and 54% (n=262), respectively. However, 64.7% (n=314) had never had the experience of on-call shadowing during their orthopedic rotations; 33% (n=160) and 37.1% (180) had received no "trauma meetings" or "OR" sessions, respectively, and only 21.9% (n=106) had attended more than five clinics. The vast majority 56.1% (n=272) reported receiving no formative assessment during their rotations (Table 2).

**Table 2:** Orthopedic curriculum assessment (n=485)

Variables	Values, n (%)
Duration of orthopedic rotation(s)	
<1 week	46 (9.5%)
1-2 weeks	136 (28%)
3-4 weeks	144 (29.7%)
>4 weeks	159 (32.8%)
Number of orthopedic lectures received	
0	12 (2.5%)
1-5	124 (25.6%)
6-10	84 (17.3%)
>10	265 (54.6%)
Number of orthopedic bedside or informal small-group teaching sessions received	
0	52 (10.7%)
1-5	276 (56.9%)
6-10	113 (23.3%)
>10	44 (9.1%)
Number of orthopedic or trauma meetings attended	
0	160 (33%)
1-5	262 (54%)
6-10	49 (10.1%)
>10	14 (2.9%)
Number of orthopedic clinics attended	
0	102 (21%)
1-5	277 (57.1)
6-10	77 (15.9%)
>10	29 (6.0%)
Number of orthopedic OR sessions attended	
0	180 (37.1%)
1-5	233 (48%)
6-10	57 (11.8%)
>10	15 (3.1%)
Number of orthopedic shifts spent shadowing on-call team	
0	314 (64.7%)
1-5	128 (26.4%)
6-10	31 (6.4%)
>10	12 (2.5%)
Received any formative assessments in orthopedics	
Yes	213 (43.9%)
No	272 (56.1%)
Orthopedics undergraduate training experience rating	
Poor	39 (8%)
Less than adequate	61 (12.6%)
Adequate	150 (30.9%)
Good	174 (35.9%)
Excellent	61 (12.6%)

Competency assessment

In general, on a scale of 1 to 10, the respondents were most confident in performing knee examinations (mean: 6.6 ±2.61), while they were least confident in managing emergency orthopedic cases (mean: 5.5 ±2.60).

**Figure 2:**
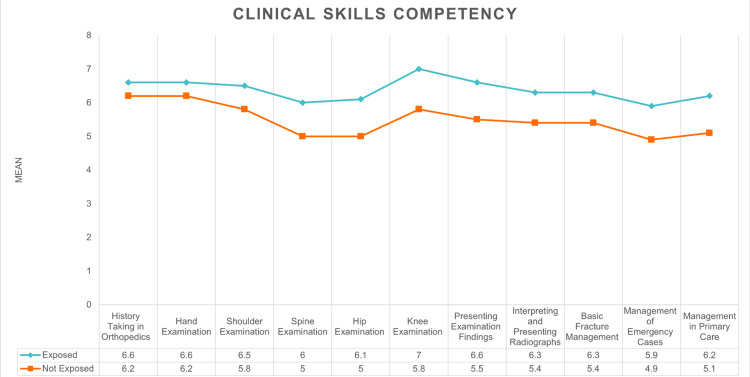
Histogram illustrating competence assessment in relation to orthopedic curriculum exposure

As shown in Figure 2, the difference between those who had attended an orthopedic rotation (mean: 7.0 ±2.38) and those who had not (mean: 5.8 ±2.8) demonstrated the widest gap in skill set in the knee examination section. Shoulder and hand examinations showed the lowest difference in mean values with a difference of 0.7 and 0.4, respectively (Table 3).

**Table 3:** Competence assessment in relation to orthopedic curriculum exposure SD: standard deviation

Variable		N	Mean ±SD	P-value
History taking in orthopedics	Yes	485	6.6 ±2.31	0.052
	No	309	6.2 ±2.43	
Hand examination	Yes	485	6.6 ±2.36	0.018
	No	309	6.2 ±2.37	
Shoulder examination	Yes	485	6.5 ±2.37	<0.001
	No	309	5.8 ±2.42	
Spine examination	Yes	485	6.0 ±2.58	<0.001
	No	309	5.0 ±2.57	
Hip examination	Yes	485	6.1 ±2.45	<0.001
	No	309	5.0 ±2.60	
Knee examination	Yes	485	7.0 ±2.38	<0.001
	No	309	5.8 ±2.80	
Presenting examination findings	Yes	485	6.6 ±2.42	<0.001
	No	309	5.5 ±2.60	
Interpreting and presenting radiographs	Yes	485	6.3 ±2.35	<0.001
	No	309	5.4 ±2.43	
Basic fracture management	Yes	485	6.3 ±2.38	<0.001
	No	309	5.4 ±2.69	
Management of emergency patients in orthopedics	Yes	485	5.9 ±2.52	<0.001
	No	309	4.9 ±2.61	
Management of primary care patients in orthopedics	Yes	485	6.2 ±2.46	<0.001
	No	309	5.1 ±2.50	

History Taking

Subjective competence was highest (mean: 8.925 ±1.299) in students who had received more than four weeks of orthopedic rotation and attended six clinics or more. Among students who had received three to four weeks of orthopedic rotation, those who had received formative assessment showed a higher degree of competence (mean: 7.113 ±2.128) than those who had not (mean: 6.244 ±2.094). The lowest perceived competence was found in those whose rotations were two weeks or less and those who had not received any form of formative assessment (mean: 5.512 ±2.51).

Presenting Examination Findings

The highest subjective skill (mean: 8.143 ±1.818) was in students who had taken three weeks or more of orthopedic rotation and also attended six or more clinics. Additionally, among those students who had received three weeks or more of orthopedic rotation and five or fewer clinics, the subsegment that had undergone formative assessment had a greater level of competency (mean: 7.203 ±2.257) compared to those who had not (mean: 6.110 ±2.082). Individuals whose rotations lasted two weeks or less had the lowest perceived competence (mean: 6.077 ±2.641).

Interpreting and Presenting Radiographs

Students with the highest scores (mean: 7.631 ±2.093) had completed an orthopedic rotation of three or more weeks and attended six or more clinics. The students who had received formative evaluation had a greater level of competence (mean: 6.959 ±2.064) among those who had completed three weeks or more of orthopedic rotation and attended five or fewer clinics. The group with rotations lasting two weeks or less had the lowest perceived competence (mean: 5.813 ±2.489).

Managing Orthopedic Cases in Primary Care Settings

Students who had completed more than four weeks of orthopedic rotation and six or more bedside sessions scored the highest (mean: 8.014 ±1.931). In contrast, the group with the lowest competence had two weeks or less of orthopedic rotation (mean: 5.462 ±2.625).

Future specialty

As shown in Table 4, only 28.46% (n=226) of the respondents stated that they would be interested in pursuing a future career in orthopedic surgery. There was a significant difference (41.1%) between the responders who had elective experience (61.4%) and those who had not (20.3%) in terms of pursuing a career in orthopedics.

**Table 4:** Plans regarding future orthopedic career

	Planning for an orthopedic career		
	Yes	No	P-value
	226 (28.46%)	568 (71.54%)	
Presence of orthopedic rotation in the curriculum			0.200
Yes	146 (30.1%)	339 (69.9%)	
No	80 (25.9%)	229 (74.1%)	
Undertaken a summer elective in orthopedics			<0.001
Yes	97 (61.4%)	61 (38.6%)	
No	129 (20.3%)	507 (79.7%)	

"New specialty" (43.40%) and "speed in treatment" (40.30%) were the two most preferred characteristics for those interested in the specialty. The two most common responses among those who had no interest in pursuing a career were a desire for "shorter work hours" (79.50%) and a less demanding "on-call schedule" (77.40%).

## Discussion

The burden of MSK disorders

In 2014, a national survey was used to investigate the patterns of costs and spending among orthopedic surgeons across the USA, which revealed an annual expenditure of 8.2 billion dollars in the USA in the field of orthopedic surgery [10]. While in Saudi Arabia, in 2019, the total cost of care for 174,225 osteoporosis-related fractures was 636 million dollars [11]. In terms of emergency orthopedic care and RTAs, it has been established that RTA is the major source of bone fractures in India, and RTA-related orthopedic injuries are the leading cause of death and disability in Ethiopia [12,13]. Nationally, in Aseer, numerous major orthopedic injuries were reported among RTA patients, and the resulting orthopedic injuries, death, and lifelong disabilities place a significant burden on economic resources [14].

Curriculum assessment and competency

The notion of shortcomings in MSK medicine coverage in modern curriculums is not new. Though no local studies have explored this topic, multiple international studies from the UK and Brazil have reached the conclusion that there is a huge room for improvement in terms of competency in MSK medicine among medical students and interns, utilizing different methods (OSCE or survey) [6,15]. Our study shows a wide range of results in each variable. Overall, the four factors that play a major role in enhancing the students' subjective level of competence are the length of rotation, the number of clinics attended, small-group sessions, and the regular conducting of formative assessments.

Length of Rotation

As in most skills assessed, history taking, presenting examination findings, management of ortho patients in primary care settings, and presenting radiographic findings, the level of competence demonstrated a direct relationship with the length of rotations. This harks back to the recommendations of the WHO between 2000-2010, termed the “Bone and Joint Decade'' highlighting that length of MSK training should be provided for six months at the minimum [5]. When looking at how this compares to the data in the literature, most studies do not follow the recommendations [5,16]. It is worth emphasizing the importance of proper timing with regard to when students should be exposed to MSK medicine, as earlier teaching in MSK medicine does not always lead to superior outcomes, in the absence of an early structured clinical exposure to patients with MSK issues [17,18].

Clinics and Bedside Sessions

It was observed that students' competency increased significantly if they attended six or more clinics/sessions, compared to their peers. Despite the certainty that quantity is by no doubt important, equal attention should be paid to the quality of the rotations, with more emphasis placed on the clinical aspect, especially since clinical experience can strengthen academic abilities, advance clinical competencies, and boost communication and confidence [19].

Formative Assessment

Formative assessment plays an important role in evaluating students’ progress, gauging their current level of competence, and allowing them to determine the areas they need to improve upon. Similarly, Black and Wiliam's review strongly suggests that improving formative assessment raises the standards of students’ performance [20]. However, it should not be at the expense of higher-quality rotations.

Orthopedics as a career choice

Clinical experience has a major role in the decision of choosing orthopedics as a future specialty, and it may be provided via summer electives or curriculum-based rotations. The data supports a significant relationship between summer electives and planning a future in orthopedics as electives could provide students with time to explore a medical or surgical specialty of their choice [21]. Regarding the impact of curriculum rotations on pursuing orthopedics as a future specialty, Bernstein et al. [22] state that the presence of MSK medicine in the curriculum was associated with a 12% higher rate of application to orthopedic surgery residency programs; however, our data showed no significant association.

Recommended solutions

As demonstrated by our data, increased duration of orthopedic rotation would make the most impact on students' experiences and perceptions. However, many solutions to improve the learning experience have been recommended in the literature. First, the peer-assisted learning (PAL) technique, in which students learn from and with each other. Perry et al. [23] have stated that MSK examination skills of final-year medical students improved and better exam results were obtained as a result of PAL's integration into the undergraduate medical curriculum. Many studies also cite the Orthopedic Surgery and Sports Medicine Interest Group (OSSMIG), an extracurricular organization that strives to increase medical students' competence and confidence in MSK medicine and generate interest in orthopedics. The University of Washington School of Medicine students have benefited from these activities, and a significant improvement in self-assessed competence and confidence was seen; moreover, the interest group's attitude toward exposure and assistance was noticeably better than that of the institutions [24]. Other recommended methods include more interuniversity-based methods and programs involving the establishment of conjoint weekly distant learning via online video forums, monthly case courses and workshops, and interuniversity's collaboration through a structured distant learning program. This could not only help students get higher exposure to different methods of learning but also build better and greater relationships and working environments with their colleagues and peers [25,26].

Limitations

This study is the largest survey conducted so far in Saudi Arabia among sixth-year medical students and interns that focus on the quantity and quality of undergraduate orthopedic rotations. Additionally, it is the first to examine students' subjective competency in critical orthopedic skills and their subjective confidence in managing orthopedic patients. However, as a retrospective study, these results show relationships rather than causality. Hence, future research should focus on evaluating students' pre- and post-course subjective and objective competency using validated objective assessment and a national survey of Saudi Arabian medical schools to examine the variations in curriculum delivery between institutions.

## Conclusions

Our survey confirms that the amount of orthopedic training provided by institutions varies, with many students having much less undergraduate orthopedic training than what is advised and desirable. However, despite these drawbacks, the current undergraduate teaching program in Saudi Arabian medical schools has certain advantages, since students who had previously participated in longer orthopedic rotations reported considerably greater levels of perceived orthopedic competence. Moreover, training that is delivered in more frequent and dynamic settings, such as small-group sessions, outpatient clinics, and formative assessment, is linked to better learning experiences and much higher subjective competence. Lastly, students and interns who had more exposure to orthopedics through curriculum and elective rotations demonstrated a stronger interest in pursuing orthopedics as a future career.
